# Minimally Invasive Repetitive UVA Irradiation along with Riboflavin Treatment Increased the Strength of Sclera Collagen Cross-Linking

**DOI:** 10.1155/2017/1324012

**Published:** 2017-12-17

**Authors:** Bo Xiao, Yanhua Chu, Hongyan Wang, Quanhong Han

**Affiliations:** Tianjin Eye Hospital, Tianjin Eye Institute, Tianjin Key Lab of Ophthalmology and Visual Science, Clinical College of Ophthalmology, Tianjin Medical University, Tianjin, China

## Abstract

**Objective:**

This study aimed to investigate the efficacy of minimally invasive repetitive UVA irradiation along with riboflavin treatment on sclera collagen cross-linking in rabbits.

**Method:**

Forty-eight healthy adult New Zealand white rabbits were randomly divided into four groups: pseudosurgery group (group I), single-irradiation group (group II), duplicate-irradiation group (group III), and triplicate-irradiation group (group IV), with 12 rabbits in each group. For the single-irradiation group, a specially made LED light source was inserted through a minimally invasive conjunctival incision to gain close contact with the sclera for irradiation, and for the repetitive irradiation groups, the above experimental procedure was repeated once or twice every other week. Biomechanical parameters of the sclera including ultimate stress (*σ*) and 8% Young's modulus (E) were compared among the groups.

**Results:**

In comparison with control group I, the ultimate stress of group II, group III, and group IV increased by 80.1%, 107.9%, and 182.1%, respectively, and their 8% Young's modulus increased by 106.1%, 159.5%, and 208.5%, respectively, one day after surgery (*P* < 0.01).

**Conclusion:**

Repetitive minimally invasive UVA irradiation with riboflavin significantly increased biomechanical strength of the sclera in the irradiated area, and biomechanical strength increased with repeated times of irradiation.

## 1. Introduction

Pathological myopia is one of the most common factors leading to visual acuity loss and blindness, with a lack of effective therapy. Many studies have shown that sclera riboflavin/UVA collagen cross-linking can effectively enhance biomechanical strength of the sclera and could potentially be one of the most promising methods for treating pathological myopia [1–4]. Previous techniques of sclera collagen cross-linking using conventional UVA irradiance devices have required relatively large conjunctival incisions, postoperative stitching and intense intraoperative pulling of the extraocular muscles, and rotation of the eye ball to expose the equatorial sclera and less effectively the posterior sclera which is most prone to damage and staphylomas [5]. These adverse factors prevented the development and application of sclera collagen cross-linking induced by riboflavin/UVA, making it difficult to implement repetitive irradiation.

In this study, we proposed a method for sclera collagen cross-linking via minimally invasive riboflavin/UVA by conducting repetitive irradiation on rabbit posterior sclera and equatorial sclera, studied the efficacy of this method, and explored whether it would be possible to enhance sclera mechanical strength by simply increasing irradiation times while keeping single-irradiation time and dose unchanged.

## 2. Methods

### 2.1. Experimental Animals and Grouping

Forty-eight healthy, clean adult New Zealand white rabbits were obtained from the Laboratory Animal Science Department of Tianjin Medical University, with males and females being randomly selected and each rabbit weighing 2.0 kg–2.5 kg. Rabbits were used in accordance with the Statement for the Use of Animals issued by the Association for Research in Vision and Ophthalmology. The protocol for treatment was approved by our institutional ethics committee on animal research (Tianjin Eye Hospital Ethics Committee). The anterior ocular segment and posterior ocular segment of these rabbits were examined to exclude possibility of ocular diseases. Rabbits were randomly assigned to four groups: pseudosurgery group (group I), single-irradiation group (group II), duplicate-irradiation group (group III), and triplicate-irradiation group (group IV). Group II, group III, and group IV, with the exception of group I, were defined as cross-linking groups, from which right eyes were selected and subjected to sclera collagen cross-linking therapy via minimally invasive riboflavin/UVA treatment. In group I, right eyes were subjected to pseudosurgery. Time interval between two consecutive irradiation treatments was one week for the repetitive irradiation groups. Each group had 12 New Zealand white rabbits, from which 6 rabbits (6 eyes) were subjected to sclera biomechanical experiments.

### 2.2. Experimental Procedure

**Figure 1:**
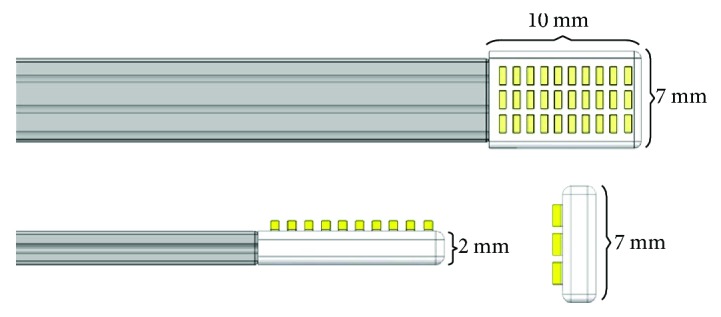
LED probe structural representation.

**Figure 2:**
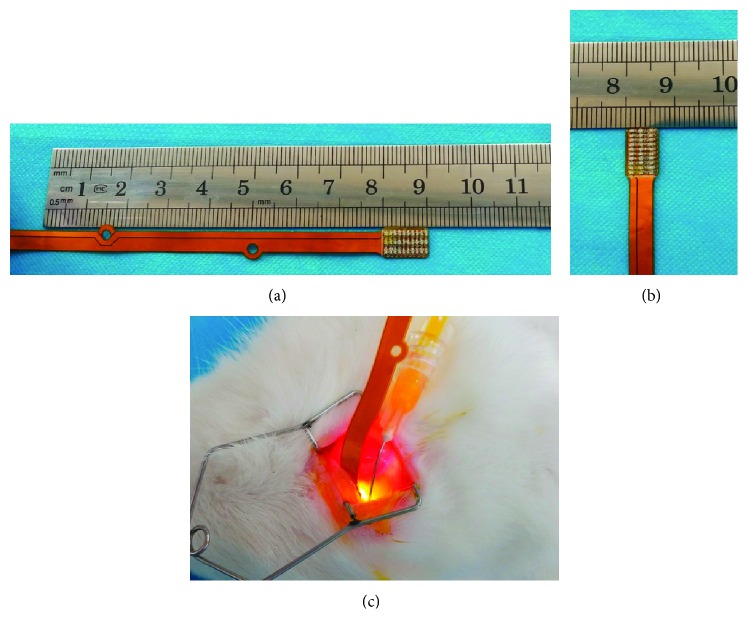
LED probe and its insertion into the surface of the sclera for irradiation.

The minimally invasive ultraviolet sclera cross-linking apparatus used in this study was developed by Tianjin Eye Hospital and Suzhou Institute of Biomedical Engineering and Technology. The central component of the apparatus consisted of a specially fabricated LED probe made of flexible material, having a size of 10 mm × 7 mm × 2 mm, and a 3 × 10 LED cold light source array attached to the probe tip (Figures 1, 2(a), and 2(b)).

**Figure 3:**
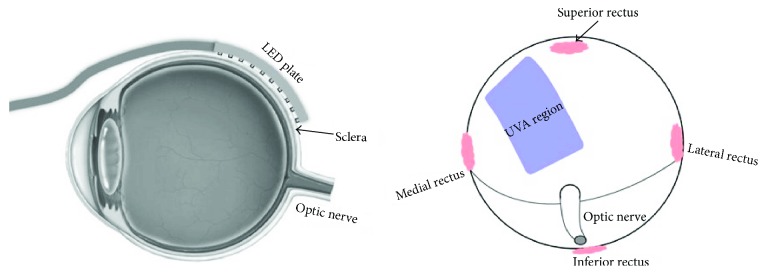
A schematic representation marking the extent of the irradiation field.

The experimental procedure for the single-irradiation group (group II) is as follows: (1) Rabbits were intraperitoneally injected with 3% sodium pentobarbital solution at a dose of 1 mL/kg, following which rabbits were in deep anesthesia. A drop of proparacaine eye drops was applied to the local area of the eye, every other minute as a surface anesthetic. (2) A superonasal conjunctival incision was made along corneal limbus to separate subconjunctival tissues, and blood on the surface of sclera was cleaned with sterile cotton swab. (3) 0.1% riboflavin solution was dropped (at a rate of 1 drop/2 min) on the sclera, with riboflavin infiltration lasting for 20 min. (4) When setting the parameters of the cross-linking device, we chose a wavelength of 370 nm (UVA) and an irradiance of 3 mW/cm^2^. The irradiation time was 30 minutes and the total UVA dose 5.4 J/cm^2^. (5) The UVA probe was slowly inserted under Tenon's capsule along the scleral surface and beyond the equator to a distance of 15 mm from the limbus allowing convenient irradiation of the posterior sclera and parts of the equatorial sclera. The LED probe was applied in direct contact with the sclera and no focusing was necessary (Figures 2(c) and 3). Upon completion of the irradiation, water was injected into the subconjunctiva, causing conjunctival swelling, to seal the conjunctival incision and erythromycin eye ointment was applied once every day for three days. For the repetitive irradiation groups (groups III and IV), the above experimental procedure was repeated once or twice every other week. For the pseudosurgery group (group I), the steps were the same as group II except that they were not irradiated.

### 2.3. Preparation of Sclera Specimens

Twenty-four hours after the first irradiation for group II or the last irradiation of groups III and IV, six experimental rabbits were randomly selected from each group and euthanized by administering intravenous injection of air in the marginal ear under deep anesthesia after intraperitoneal injection of 3% sodium pentobarbital solution at a dose of 1 mL/kg. The right eyes were enucleated: tissue around the eyeballs was carefully separated with sharp-headed surgical scissors, the eyeballs were taken out and cut open along the corneoscleral margin to remove contents of the anterior and posterior ocular segments. Subsequently, the sclera was rinsed clean with 0.9% normal saline and dried with a cotton swab. For subsequent experiments, two rectangular-shaped scleral bands with a size of approximately 3 mm × 10 mm were collected from the irradiated cross-linking area or the same nonirradiated area of group I in the posterior region and part of the equatorial region of the superonasal quadrant and stored in a moisture chamber at 4°C for biomechanical examination.

### 2.4. Biomechanical Examination

**Figure 4:**
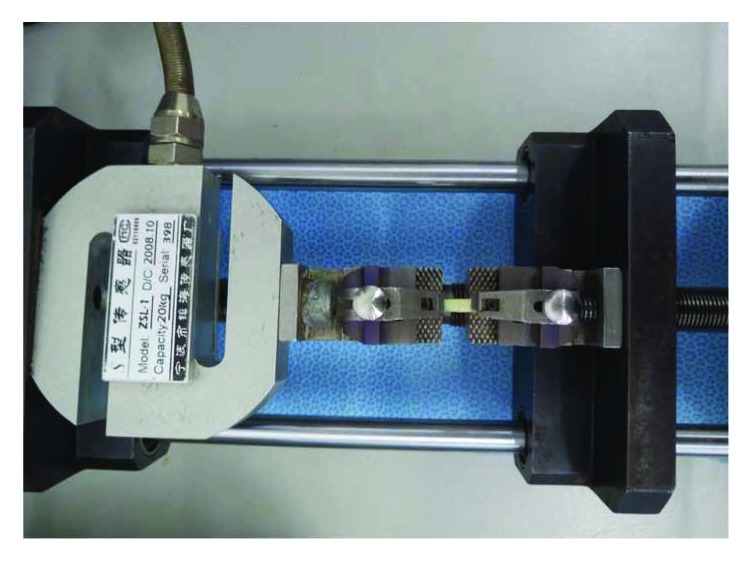
ZSL-1 biomaterial test system.

The length and thickness of each sclera specimen were measured with a Vernier caliper and micrometer, respectively. The prestretching experiment of the sclera was conducted with a biomaterial test system (Figure 4) (ZSL-1, Ningbo Zhenhai Sensor Co. Ltd.). The scleral band was horizontally clamped on a clamping apparatus and fixed to the test system for biomechanical examination. The length of the section to be stretched ranged from 4 to 6 mm, and 0.9% normal saline was sprayed on the band once every 2 min to ensure that the band would remain moist during stretching. The sclera was subjected to preloading/unloading at the same stress level, the number of cycles was set at 7, the loading speed was set at 0.5 mm/min, and the stop criterion was set to load above 0.021 N. When the load displacement curve provided by the test system gradually approached stable values, tensile failure experiments were conducted, with a loading speed of 1 mm/min to stretch the sclera until broken. The ultimate stress (*σ*) was recorded when the scleral band broke, and 8% Young's modulus was calculated, where stress = load/cross-sectional area, strain = displacement/original length, Young's modulus = stress/strain, cross-sectional area = wideness × thickness. The load-displacement data was provided by the biomaterial test system.

### 2.5. Statistical Analysis

In this study, statistical analyses were performed using SPSS 17.0 software (SPSS, USA). Data were expressed as mean ± standard deviation (mean ± SD). The comparison of biomechanical parameters in groups I–IV (ultimate stress and 8% Young's modulus) was subjected to repeated measures one-way analysis of variance (RM-ANOVA). Post hoc analysis was performed using Fisher's LSD test. *P* < 0.05 was considered statistically significant.

## 3. Results

### 3.1. Sclera Biomechanical Examination

In this study, we employed two important biomechanical indices (ultimate stress and 8% Young's modulus) that are globally accepted for evaluating viscoelastic biological tissues. When biological materials are broken, the stress per unit area is referred to as ultimate stress, which reflects the limit of external force per unit area that the biological material can withstand and indicates the magnitude of internal force per unit area of the biological material, in kilopascal (kPa). Ultimate strain refers to the ratio of length increment to initial length along the direction of stress when biological materials are broken, and it is dimensionless. 8% Young's modulus (in kPa) is the ratio of stress to strain at 8% strain, reflecting the capability of biological materials of resisting deformation.

Table 1 shows the ultimate stress of sclera among groups I–IV. There was a significant difference in ultimate stress among groups (*F* = 26.39, *P* = 0.00). In comparison with group I, the sclera ultimate stress in groups II, III, and IV increased by 80.1%, 107.9%, and 182.1%, respectively (*P* = 0.00). Similarly, 8% Young's modulus was significantly different among the groups (*F* = 35.55, *P* = 0.00). Compared to group I, 8% Young's modulus increased by 106.1%, 159.5%, and 208.5% in groups II, III, and IV, respectively (*P* = 0.00).

## 4. Discussion

Pathological myopia (usually with more than −6 D) often involves degeneration of the sclera, choroid, and retinal pigment epithelium and is accompanied by visual acuity loss [6, 7]. The pathological changes include posterior scleral staphyloma, choroidal neovascularization, retinal choroidal atrophy, streak, and peripheral retinal degeneration [8, 9]. PM is one of the leading cause of blindness and low visual acuity in East Asia as well as in other parts of the world [10–12]. Population-based surveys indicated that 7% of blindness and low visual acuity was caused by PM in Europe [13] and was as high as 12%–27% in Asia [14]. PM generates dual burdens on the psychology and physiology of patients and may lead to blindness and social impairment, making it a widespread medical problem to be resolved. It is widely accepted that PM is caused by genetic [15] as well as environmental [16, 17] factors that act on the sclera, undergoing proactive reshaping which includes diameter decrease and fiber gap enlargement of the collagenous fiber bundle in all parts of the myopic eye, particularly in the posterior sclera. This results in the decrease of normal cross-linked fibers and causes progressive thinning and expansion of the sclera and overextension of the ocular axis, thereby causing retinal fundus complications to arise which adversely affect visual acuity [18, 19].

In the last decade, a number of experimental studies have shown that riboflavin/UVA collagen cross-linking can effectively enhance biomechanical strength of sclera and is likely to become an effective therapeutic method to treat PM [1–4]. In 2004, Wollensak and Spoerl were the first to apply scleral cross-linking and to propose scleral cross-linking as a treatment to heal progressive myopia similar to the cornea with progressive keratoconus [1].

In 2005, Wollensak et al. used healthy velveteen rabbits to conduct a more in-depth study on the efficacy and safety of riboflavin/UVA collagen cross-linking, and the results showed that there was an improvement in the biomechanical parameters of rabbit sclera, but many of the retinal photoreceptor cells and pigment epithelial cells were lost [20]. In order to investigate and solve the problem of retinal cell loss, Wollensak et al. made an adjustment to experimental parameters in 2009, fixing the energy density of UVA to 5.4 J/cm^2^, and the results showed that the riboflavin/UVA collagen cross-linking method could significantly enhance biomechanical strength of rabbit sclera without resulting in any apparent pathological damage to tissues in the eyes [21]. This demonstrates that energy density of UVA has a considerable effect on the safety of riboflavin/UVA collagen cross-linking method. Extremely high UVA energy density results in significant loss of retinal cells, and extremely low energy density is unable to effectively improve cross-linking of the sclera. In 2014, Yali et al. conducted riboflavin/UVA collagen cross-linking on New Zealand rabbits and discovered that biomechanical strength of rabbit sclera was significantly augmented [22]. In 2016, Dotan et al. found that the riboflavin/UVA collagen cross-linking method could effectively prevent elongation of ocular axis [23]. In 2013, Choi et al. demonstrated riboflavin/UVA collagen cross-linking in human cornea-sclera and reported that collagen fibers in sclera and cornea were aligned tightly and showed an increase in diameter as well as thickness [24]. These reports provide reliable evidence demonstrating biomechanical and ultrastructural changes of sclera during collagen cross-linking.

Nowadays, corneal collagen cross-linking has been used in clinics, but cross-linking in sclera has not yet been utilized in clinical settings because of several obstacles that need to be overcome, particularly, the inconvenience of utilizing ultraviolet radiation to irradiate sclera. In previous studies, UVA irradiation apparatuses were employed to remotely irradiate target scleral region. In the irradiation process, eyes had to be stretched to expose the target scleral region and irradiation was largely focused on the equatorial area, thereby inducing possible mechanical injury to ocular tissues, and this approach made it impossible to fully expose the posterior sclera that is prone to develop staphyloma [5]. These adverse factors prevent the development and application of sclera collagen cross-linking induced by riboflavin/UVA; moreover, it is difficult to implement repetitive irradiation.

In order to overcome the many adverse factors encountered in the past, such as complexity of operating riboflavin/UVA sclera collagen cross-linking, large lesions, difficulty in irradiating the posterior sclera, and difficulty in administering repetitive irradiation, this study proposed a minimally invasive riboflavin/UVA sclera collagen cross-linking method. Based on the experimental parameters reported in past studies, we used living rabbits as experimental subjects and inserted a light-source probe beneath the conjunctiva, directly on the surface of the sclera. The probe was in close contact with the posterior and equatorial sclera emitting radiation to induce effective sclera collagen cross-linking. A syringe with a blunt needle allowed an almost continuous supply of the riboflavin photosensitizer solution during the irradiation.

This study demonstrated that repetitive minimally invasive irradiation could significantly increase biomechanical strength of sclera in irradiated areas. Compared to the control group, the ultimate stress one day after surgery was increased by 80.1%, 107.9%, and 182.1% in the single-irradiation group, duplicate-irradiation group, and triplicate-irradiation group, respectively. 8% Young's modulus was increased by 106.1%, 159.5%, and 208.5%, respectively.

The experimental results of this study indicated that minimally invasive irradiation of rabbit posterior sclera could significantly enhance biomechanical strength of sclera in the irradiated area. With increase of the irradiation times, the biomechanical strength of sclera in the irradiated area increased.

In summary, the cross-linking method proposed in this study was unique since only a small lesion was created and the eyeball tissues had not been stretched to get access to the posterior sclera. According to the cross-linking method of this study, the light-source probe could be inserted beneath the conjunctiva to implement effective irradiation to the posterior sclera which conventional irradiation devices do not provide. Moreover, after irradiation surgery, the conjunctival incision could be sealed by conjunctival edema through subconjunctival water injection, achieving a minimally invasive therapy, facilitating studies on the promotional effect of repetitive irradiation on biomechanical strength of the sclera, and enhancing the possibility of its use in future clinical settings.

## Figures and Tables

**Table 1:** Biomechanical parameters ($\bar{x}\pm S$, *n* = 12).

Group	Group I	Group II	Group III	Group IV
Ultimate stress *σ* (MPa)	3.52 ± 1.38	6.34 ± 1.67^∗^	7.32 ± 1.83^∗^	9.93 ± 2.17^∗^
8% Young's modulus E (MPa)	13.36 ± 3.84	27.54 ± 6.34^∗^	34.67 ± 7.24^∗^	41.21 ± 9.23^∗^

Note: compared to group I, the ultimate stress and 8% Young's modulus of groups II–IV were significantly different. ^∗^*P* < 0.01 (one-way ANOVA, LSD *t*-test).
